# Mitigation role of physical exercise participation in the relationship between blood cadmium and sleep disturbance: a cross-sectional study

**DOI:** 10.1186/s12889-023-16358-4

**Published:** 2023-07-31

**Authors:** Yanwei You, Yuquan Chen, Yangchang Zhang, Qi Zhang, Yaohui Yu, Qiang Cao

**Affiliations:** 1 Division of Sports Science & Physical Education, Tsinghua University, Beijing, 100084 China; 2 Institute of Medical Information/Medical Library, Chinese Academy of Medical Sciences & Peking Union Medical College, Beijing, 100020 China; 3 Department of Epidemiology and Biostatistics, School of Public Health, Capital Medical University, Beijing, 100169 China; 4 Undergraduate Department, Taishan University, Taian, 250111 China; 5 School of Public Health, North China University of Science and Technology, Tangshan, 063210 Hebei China; 6 Department of Earth Sciences, Kunming University of Science and Technology, Kunming, 650093 China; 7 School of Pharmacy, Macau University of Science and Technology, Macau, Macau, 999078 China

**Keywords:** Heavy metal, Cadmium, Physical exercise, NHANES, Sleep disturbance, Cross-sectional study

## Abstract

The neurotoxicity of heavy metals received increasingly attention in recent years. Sleeping is regulated and coordinated by nervous system, however, the health hazard of heavy metal like cadmium (Cd) exposure on sleep health remained unknown. Rescue strategies like physical exercise (PE) has emerged to mitigate such influence. An epidemiological design with cross-sectional data from National Health and Nutrition Examination Survey 2007–2010 was applied. The relationship between three blood heavy metals [cadmium (Cd), lead (Pb), mercury (Hg)] and sleep disturbance was analyzed. A total of 8,751 participants were finally included in and the weighted participants were 330,239,463. Weighted quantile sum (WQS) regression indicated that mixed blood metals were positively related to risk of sleep disturbance and the mixture effect of exposure to heavy metals was mainly attributable to Cd (89.1%). Weighted logistic regression showed a significant positive association between the highest quartile of blood Cd and sleep disturbance [(OR (95% CI)): 1.191 (1.014,1.400), p = 0.036] in the fully adjusted model, while no association was found under Pb and Hg exposure. In the association between Q3 and Q4 level of blood Cd and sleep disturbance, moderate-to-vigorous physical exercise group had lower risks than none and low exercise group. In the restricted cubic spline model, it was also verified that higher PE participation was associated with the lowest incidence of sleep disturbance with the increment in Cd concentration. Our study suggested that both policy makers and the public should minimize heavy metal exposure. Moreover, conducting moderate to vigorous physical exercise is a protecting factor to mitigate Cd’s influence on sleep health.

**Supplementary Information:**

The online version contains supplementary material available at 10.1186/s12889-023-16358-4.

## Introduction

Environmental toxic metals, including cadmium (Cd), lead (Pb) and mercury (Hg), have recently received intense attention in consideration of their widespread exposure and adverse health effects [1–3]. Heavy metals, which are widely used in electronics, optics, as well as medical industries, can affect the human system through ingestion of contaminated water and food, inhalation of ambient air, and skin contact. Compared with the broad consensus of metals on cardiovascular or metabolism system, metal toxicity on the function and progression of multiple neurological diseases (such as sleep disturbance, stroke, Alzheimer’s disease) is emerging topics [4] and has not been fully examined.

On the basis of the close relationship between neurological and brain health with sleep, the influence of metal pollutants on sleep health is another research topic worth exploring. As a rising public health issue, poor sleep and sleep disturbance is more common in modern society and one third of the US general population self-reported troubling sleeping experiences [5]. Relative to other toxic metals, more research has been done on lead and cadmium, perhaps attributed to their significant morbidity and mortality. One previous case–control study found that higher blood lead and cadmium concentrations were significantly associated with obstructive sleep apnea (OSA, one of a sleep breathing disorder) [6]. There are mainly two pathways that environmental pollutants can affect the brain as well as sleep: one is through food intake and the metabolic system, and another is through the circulation system and the blood-brain barrier (BBB) [7, 8].

Although there were hypotheses and concerns about sleep troubles due to metal exposure, the existing research was hard to fully explain the etiology of sleep related disorders, and epidemiological study on the nationally population remained scarce. Over recent years, one cross-sectional research using nationwide samples from the National Health and Nutrition Examination Surveys (NHANES) showed that higher urinary arsenic level was related to frequent night awakenings [9], and higher urinary antimony concentration was associated with sleep health concerns including OSA [10]. Nevertheless, to the best of our knowledge, limited evidence has explored the relationship between heavy metal (including Cd and Pb) exposure and sleep disturbance.

As a rescue strategy, physical exercise (PE) has been reported to improve sleep quality and efficiency. In recent years, plentiful trials focused on the benefits of exercise on sleep health and a number of systematic reviews and meta-analyses were conducted to verify the efficiency of exercise on sleep related disorders [11–13]. Abundant evidence suggested that there are biological and psychosocial mechanisms induced by exercise [14–16], which may improve sleep symptoms and quality of life. Given that PE can induce beneficial effects on sleep health, while exposure to heavy metal was harmful on sleep disturbance, these facts, led us to propose whether PE could mitigate the negative effects of metal exposure. The major weaknesses of previous studies included shortage of specific metal exposure (such as Cd) assessments, as well as the lack of data on confounders (e.g., body mass index, chronic diseases). Additionally, the joint effect of environmental exposure and lifestyle factor including PE on sleep disturbance was unknown.

Using nationwide–represented data from the NHANES, we aimed to explore the association between the exposure of blood metal with sleep disturbance as well as the mitigation effects of exercise. We hypothesized that toxic metal exposure was positively associated with sleep disturbance and PE can alleviate this association. Collectively, this cross-sectional study aimed to: (i) investigate the associations between metal exposure and sleep disturbance; (ii) identify the most relevant metal on sleep disturbance and further explore the influence of different subgroups; (iii) explore the mitigation effect of PE on relationship to the associations mentioned above.

## Methods

### Study population

All data used in this current research were obtained from the National Health and Nutrition Examination Survey, which was implemented by the National Center for Health Statistics. In order to reflect the noninstitutionalized civilian population residing in the United States, NHANES was designed as a stratified, multistage probability samples through a complex statistical process to reflect all resident population information. The interview of NHANES covered demographic, socioeconomic, physiological and biochemical indexes as well as other health-related issues. Unique sampling weight was assigned to each participant and multidimensional index such as interviewed weights (interviews in the home), mobile examination center (MEC) weights, or blood metals should be carefully applied in different situations. Sample information and processing methods from NHANES for epidemiological and health related research can be publicly achieved from the online website (https://www.cdc.gov/nchs/nhanes/index.htm). Written informed consent was obtained from each participant in NHANES and the survey protocol was approved by the National Center for Health Statistics (NCHS) Research Ethics Review Board.

**Fig. 1:**
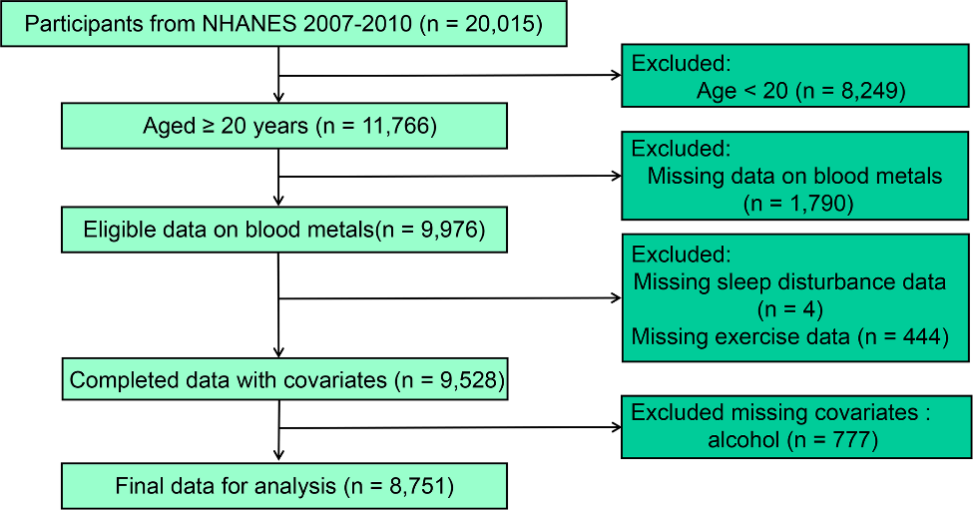
The flow chart of participant selection using the National Health and Nutrition Examination Survey data 2007–2010

We aggregated two year cycles’ (four years) data from NHANES (2007–2010). Among the 20,015 participants in the total sample, adult’s samples (n = 11,766) were used for further research. A total of 2,238 samples were excluded due to lack of information of blood metal, sleep status, and information of physical exercise behavior. Participants without covariates’ data (n = 777) were further excluded. Finally, 8,751 adults were enrolled in for analysis (Fig. 1).

### Blood metals exposure assessment

Blood metals’ sampling weights were applied to analyze these data properly. In NHANES 2007–2008 and 2009–2010, three indexes of heavy metal including Cadmium (Cd), Lead (Pb), and Mercury (Hg) were evaluated by the inductively coupled plasma-mass spectrometer (ICP-MS). Whole blood specimens were processed and stored to the Division of Laboratory Sciences and Vials were stored under frozen (–30 °C) conditions until they were shipped to National Center for Environmental Health for testing. Details of laboratory processing and quality assurance were available in the survey website of the two years’ cycle [17, 18]. Lower limits of detection (LLOD) for each metal, as well as the number and percentage of samples below the LLOD were presented in detection rate. In cases where the result was below the limit of detection, the value below the LLOD was imputed as the detection limit divided by the square root of 2.

### Ascertainment of sleep disturbance outcomes

In the present analysis, the identification of sleep disturbance cases was conducted through interview and self-reported sleeping status in the NHANES 2007–2010 survey. Referring to the interview guideline, cases of sleep disturbance were defined by utilizing a computer-assisted personal interviewing system by trained interviewers in the home as the following question “Have you ever told a doctor or other health professional that you have trouble sleeping?”, and this classification method was used by prior published studies [19, 20].

### Ascertainment of physical exercise exposure

Physical exercise (PE) information was obtained during the household interviews utilizing the Physical Activity Questionnaire. PE was defined as leisure time or recreational physical engagement (including sports, fitness and other recreational activities) in the NHANES. Before NHANES 2007, it was difficult to calculate volume of exercising in detail, while the NHANES physical exercise questionnaire changed after 2007, and low, moderate and vigorous recreational activity can be assessed by calculating the metabolic equivalent of task (MET). Days and minutes of exercise in a typical week was extracted to calculate the total time (counted in minutes). Subsequently, the MET value for PE was obtained by the following formula: PE (MET-minutes per week) = MET (vigorous as 8 and moderate as 4) × weekly frequency ×duration of each recreational activity, the detailed calculation method was described elsewhere [21]. According to the previous literature, PE status was categorized into three level (none, low, and moderate-to-vigorous) [22].

.

### Covariates determination

Referring to previous literature, relevant covariates were considered as potential confounding factors in our analysis [21, 23], including socio-demographic characteristics (age, gender, race/ethnicity, marital status, education, poverty income ratio), lifestyle factors [body mass index (BMI), smoking status, alcohol use] and chronic disease conditions [diabetes mellitus (DM) and cardiovascular disease (CVD)]. Age was grouped into < 40, [40, 60), and ≥ 60. Gender was dichotomized into male and female. Education was categorized into three groups (below high school, high school, and college or above). Race/ethnicity was categorized as non-Hispanic white, non-Hispanic black, Mexican American and others. Marital statuses were grouped into married/living with partner, never married, widowed/ divorced. Family poverty-to-income ratio was divided into three groups (< 1, [1,3), and ≥ 3). BMI was calculated as body weight in kilograms divided by meters squared and categorized as < 25, [25, 30), and ≥ 30. According to previous literature [24, 25], smoking status was categorized into never, former, and current; and alcohol use status was categorized into never, moderate drinkers, heavy drinkers.

### Statistical analysis

Due to the probability sampling design, sample weights, strata, and primary sampling units of NHANES program, all analysis strategy in this study accounted for the complex survey design, survey non-response, and post-stratification adjustment. Weighted percentages of variables were reported and weighted chi-square test was used to show the differences in baseline characteristics. Further, survey-weighted logistic regression model were performed to estimate odds ratios and 95% CI for associations between heavy metal exposure and the sleep disturbance. There were three modeling strategies applied in this study: [1] single-metal analysis, which used separate model for each blood metal; [2] weighted quantile sum (WQS) analysis, in order to detect the importance of each metal and evaluating the mixture effect; [3] sensitive analysis using two-metal or multiple-metal model detecting blood metals simultaneously. For the weighted logistic regression, the crude model with adjusted for no covariates. Age, gender, race/ethnicity were adjusted in Model 1. Fully adjusted covariates, including BMI, marital status, education, poverty income ratio, smoking status, alcohol use and chronic diseases were adjusted in Model 2.

Referring to several previous studies [26, 27], we assumed that the direction of the association between metal exposures (Cd, Pb, and Hg) and sleep disturbances was positive. In order to verify the assumption, a regression analysis was conducted between individual blood metals and sleep disturbances. If the assumption held, the WQS regression would be applied to explore multi-metal exposures and sleep disturbance risks. For the WQS model, in detail, all blood metals were combined into a WQS index which was generated through bootstrap sampling and then scored into quartiles. The weights of each metal were constrained between 0 and 1 and can identify important (highly weighted) metals. Blood metals were randomly split into training and validation sets in WQS model (rate was 40:60), where the training set was used to estimate variable weights via 1000 bootstrap samplings and the validation set was used to test the significance of the mixture. In the WQS regression, covariates were adjusted using the fully adjusted model (Model 2).

**Fig. 2:**
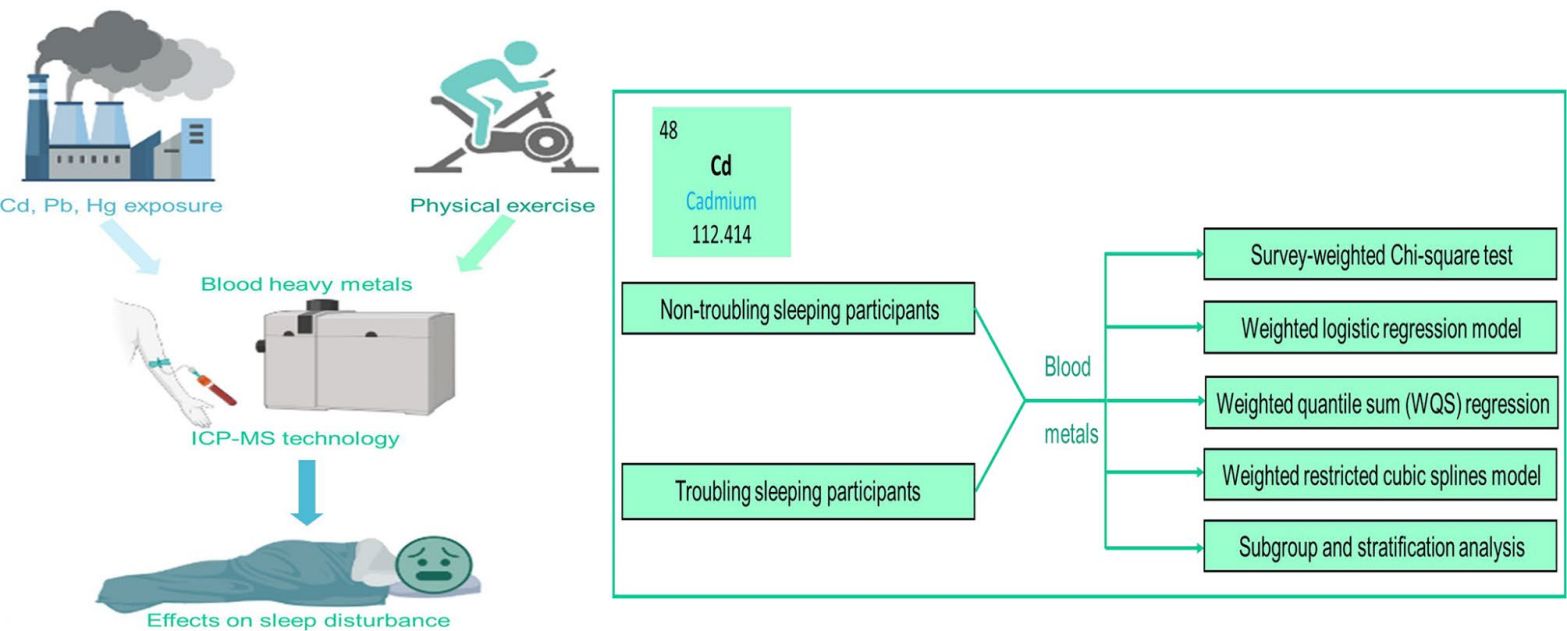
Brief introduction of the research design and analytical procedures

Based on the results of weighted logistic regression model, significant relationship between specific metal and sleep disturbance was further examined. Dose-response relationship between specific metal exposure and sleep disturbance was estimated using weighted restricted cubic spline (RCS), and 3 knots were established in our models [28]. Additionally, subgroup analyses were conducted to explore the influence of different PE levels on the relationship between heavy metal and sleep disturbance. An illustration of the research design and analytical procedures can be seen in Fig. 2. All analyses were performed using R software (version 4.2.0) and P value less than 0.05 was regarded as statistically significant.

## Results

### Basic characteristics of the study participants

We included 8,751 adults aged 20 years or older in the present analyses and the weighted participants were 330,239,463. The weighted prevalence of sleep disturbance was 25.2%. The blood metal concentration was categorized into four quartiles [Cd: Q1 (< 0.22 µg/l), Q2 (0.25–0.35 µg/l), Q3 (0.35–0.62 µg/l), Q4 (> 0.62 µg/l); Pb: Q1 (< 0.89 µg/dl), Q2 (0.89–1.37 µg/dl), Q3 (1.37–2.13 µg/dl), Q4 (> 2.13 µg/dl); Hg: Q1 (< 0.49 µg/l), Q2 (0.49–0.88 µg/l), Q3 (0.88–1.68 µg/l), Q4 (> 1.68 µg/l)]. The median age was 50 years, and 47.73% of them were men. Over half of the total participants were more likely to be non-Hispanic white and married, with a higher education level (College or above) and higher income, and less likely to be diabetes mellitus or cardiovascular patients. Detailed characteristics of participants in this study is shown in Table 1.

**Table 1:** Weighted characteristics of participants in the NHANES (2007–2010) by sleep disturbance

Variable	All participants	Non-troubling sleeping	Troubling sleeping	*P-value*
Age (year)	50.45 ± 0.35	46.38 ± 0.40	50.58 ± 0.36	< 0.001
BMI(kg/m^2^)	28.89 ± 0.10	28.52 ± 0.13	29.96 ± 0.17	< 0.001
Blood Cd (µg/l)	0.52 ± 0.01	0.50 ± 0.01	0.58 ± 0.02	< 0.001
Blood Pb (µg/dl)	1.61 ± 0.03	1.61 ± 0.03	1.59 ± 0.05	0.629
Blood Hg (µg/l)	1.63 ± 0.07	1.70 ± 0.08	1.41 ± 0.05	< 0.001
Age				< 0.001
< 40	35.40	39.00	24.75	
[40, 60)	39.83	37.35	47.15	
≥ 60	24.77	23.64	28.10	
BMI(kg/m^2^)				< 0.001
< 25	30.05	31.41	26.05	
[25, 30)	34.03	34.54	32.53	
≥ 30	35.91	34.05	41.42	
Gender				< 0.001
Male	47.73	50.48	39.60	
Female	52.27	49.52	60.40	
Race/ethnicity				< 0.001
non-Hispanic White	71.55	69.14	78.68	
non-Hispanic Black	10.18	10.28	9.89	
Mexican American	8.23	9.46	4.58	
Other Race/ethnicity	10.04	11.12	6.85	
Marital status				< 0.001
Never married	15.93	16.71	13.61	
Married/living with partner	65.30	66.68	61.21	
Widowed/ divorced	18.77	16.61	25.18	
Poverty income ratio.				0.293
< 1	13.57	13.14	14.84	
[1,3)	35.68	36.05	34.57	
≥ 3	50.75	50.81	50.59	
Education				0.344
Below high school	5.95	6.07	5.60	
High school	37.30	36.76	38.90	
College or above	56.75	57.17	55.50	
Smoking status				< 0.001
None	53.34	55.82	46.00	
Former smoking	24.98	23.68	28.81	
Current smoking	21.68	20.50	25.19	
Alcohol use				0.036
None	27.36	26.68	29.36	
Moderate alcohol use	51.18	51.01	51.68	
High alcohol use	21.46	22.30	18.96	
Physical exercise				< 0.001
None	49.86	48.07	55.17	
Low	17.51	17.62	17.19	
Moderate to vigorous	32.63	34.31	27.64	
DM				< 0.001
No	84.45	85.98	79.96	
Yes	15.55	14.02	20.04	
CVD				< 0.001
No	91.19	93.00	85.83	
Yes	8.81	7.00	14.17	
Blood Cd (µg/l)				< 0.001
Q1 (< 0.22)	26.09	27.09	23.12	
Q2 (0.25–0.35)	27.54	28.24	25.47	
Q3 (0.35–0.62)	23.02	22.71	23.93	
Q4 (> 0.62)	23.35	21.96	27.47	
Blood Pb (µg/dl)				0.766
Q1 (< 0.89)	27.84	28.23	26.70	
Q2 (0.89–1.37)	27.06	26.73	28.03	
Q3 (1.37–2.13)	24.21	24.15	24.38	
Q4 (> 2.13)	20.89	20.89	20.90	
Blood Hg (µg/l)				0.033
Q1 (< 0.49)	23.66	22.84	26.09	
Q2 (0.49–0.88)	24.14	24.18	24.03	
Q3 (0.88–1.68)	24.85	24.57	25.69	
Q4 (> 1.68)	27.34	28.41	24.19	

Notes: % for categorical variables. Abbreviations: NHANES, National Health and Nutrition Examination Survey; BMI, body mass index; PIR, poverty income ratio; Cd, cadmium; Pb, lead; Hg, mercury; DM, diabetes mellitus; CVD, cardiovascular diseases. For categorical variables, data was presented as survey-weighted percentage, P-value was calculated by survey-weighted Chi-square test; For continuous variables, data was presented as survey-weighted mean ± SE (standard error), P-value was calculated by survey-weighted linear regression

### Associations between heavy metal exposure and sleep disturbance

The associations between blood Cd, Pb, Hg level and sleep disturbance are shown in Table 2 and Supplementary Table 1. Participants were categorized according to interquartile range of Cd concentration with Q1 level as reference. A positive association between Cd and sleep disturbance was identified in Q4 level for all three models [Crude Model: OR (95% CI) as 1.466 (1.270,1.692), p < 0.001; Model 1: OR (95% CI) as 1.233 (1.058,1.436), p = 0.009; and Model 2: OR (95% CI) as 1.191 (1.014,1.400), p = 0.036]. Although a positive trend was identified, no statistically significant relationship was found between Pb, Hg and risk of sleep disturbance after adjusting for covariates in the fully adjusted model (Model 2).

**Table 2:** Weighted logistic regression results for the relationship between blood Cd and sleep disturbance

		Crude model			Model 1			Model 2	
		OR (95% CI)	*P-value*		OR (95% CI)	*P-value*		OR (95% CI)	*P-value*
Cd (µg/l)									
Q1 (< 0.22)		Ref.			Ref.			Ref.	
Q2 (0.25–0.35)		1.057(0.903,1.238)	0.479		0.927(0.796,1.080)	0.317		0.961(0.821,1.123)	0.582
Q3 (0.35–0.62)		1.235(1.024,1.489)	0.028		1.011(0.824,1.240)	0.913		1.012(0.808,1.267)	0.909
Q4 (> 0.62)		1.466(1.270,1.692)	< 0.001		1.233(1.058,1.436)	0.009		1.191(1.014,1.400)	0.036
Trend test			< 0.001			0.012			0.019

Notes: Crude model, no covariates were adjusted. Model 1, age, gender, race/ethnicity were adjusted. Model 2, age, gender, race/ethnicity, body mass index, marital status, education, poverty income ratio, smoking status, alcohol use, and chronic diseases were adjusted

Pearson correlations among blood metals were displayed as a correlation matrix in Supplementary Fig. 1(A). Considering that a unidirectional (positive) assumption was already verified, a multivariable WQS model was applied. Results showed that mixed blood metals were positively related to risk of sleep disturbance. The mixture effect of exposure to heavy metals was mainly attributable to Cd (89.1%), followed by Hg (10.8%) and Pb (0.1%) (Supplementary Fig. 1(B)), which showed that Cd exposure was the most important influencing factor of sleep disturbance in this study. Moreover, the smooth curve fitting method was used to describe the relationship between WQS index and risks of sleep disturbance (Supplementary Fig. 1(C)).

To test the robustness of the correlation between Cd level and sleep disturbance, we also performed two-metal and multi-metal regression models (incorporating multiple metals simultaneously). After adjustment for all covariates, we estimated the association between Cd exposure and sleep disturbance. The association persisted in multi-metal models and Cd in the Q4 level was positively correlated with the risk of sleep disturbance (Table 3).

**Table 3:** Weighted logistic regression results for the two-metal model and multi-metal model on the relationship between blood Cd and sleep disturbance

Heavy metal		Cd (µg/L)			
		Q1 (< 0.25)	Q2 (0.25–0.35)	Q3 (0.35–0.62)	Q4 (> 0.62)
Two-metal model					
Cd	+ Pb	Ref.	0.971(0.821,1.149)	1.035(0.816,1.311)	1.240(1.029,1.494)
	+ Hg	Ref.	0.962(0.813,1.138)	1.012(0.795,1.288)	1.180(0.991,1.406)
Multi-metal model					
Cd	+ Pb + Hg	Ref.	0.971(0.801,1.176)	1.029(0.790,1.340)	1.218(0.984,1.509)

Notes: Fully adjusted model were used. Age, gender, race/ethnicity, body mass index, marital status, education, poverty income ratio, alcohol use, and chronic diseases were adjusted

### Stratified analysis

Based on above results, Supplementary Table 2 demonstrates the association between blood Cd and sleep disturbance by stratified analysis. It was found that females [OR (95% CI): 1.509(1.185,1.923), p = 0.002] had a greater risk of sleep disturbance than males. Additionally, participants who were non-Hispanic White [OR (95% CI): 1.419(1.189,1.694), p < 0.001], non-Hispanic Black [OR (95% CI): 1.501(1.001,2.251), p = 0.049], or Mexican American [OR (95% CI): 2.383(1.490,3.810), p < 0.001] were more vulnerable to cadmium exposure with sleep disturbance. Moreover, population younger than 60 years old, BMI < 30 kg/m^2^, education level below high school or above college, unmarried, worse family income, smokers, alcohol consumption, diagnosed with cardiovascular diseases were more likely to suffer from sleep disturbance with the increment in blood Cd concentrations.

### Potential mitigation role of exercise in the relationship between blood Cd and sleep disturbance

The weighted logistic regression analyses showed significant negative associations between moderate to vigorous physical exercise (MVPE) and sleep disturbance (Table 4). In Crude Model, (MVPE) vs. (None), OR (95% CI): 0.702 (0.598,0.824), p < 0.001; In Model 1, (MVPE) vs. (None), OR (95% CI): 0.736 (0.626,0.865), p < 0.001; In Model 2, (MVPE) vs. (None), OR (95% CI): 0.804 (0.660,0.981), p = 0.034. However, no significant association was found between low level exercise and sleep disturbance.

**Table 4:** Weighted logistic regression results for physical exercise with sleep disturbance

		Crude model			Model 1			Model 2	
		OR (95% CI)	*P-value*		OR (95% CI)	*P-value*		OR (95% CI)	*P-value*
Physical exercise									
None		Reference			Reference			Reference	
Low		0.850(0.704,1.027)	0.089		0.848(0.710,1.013)	0.068		0.910(0.726,1.140)	0.378
Moderate to vigorous		0.702(0.598,0.824)	< 0.001		0.736(0.626,0.865)	< 0.001		0.804(0.660,0.981)	0.034

Notes: Crude model, no covariates were adjusted. Model 1, age, gender, race/ethnicity were adjusted. Model 2, age, gender, race/ethnicity, body mass index, marital status, education, poverty income ratio, smoking status, alcohol use, and chronic diseases were adjusted

**Fig. 3:**
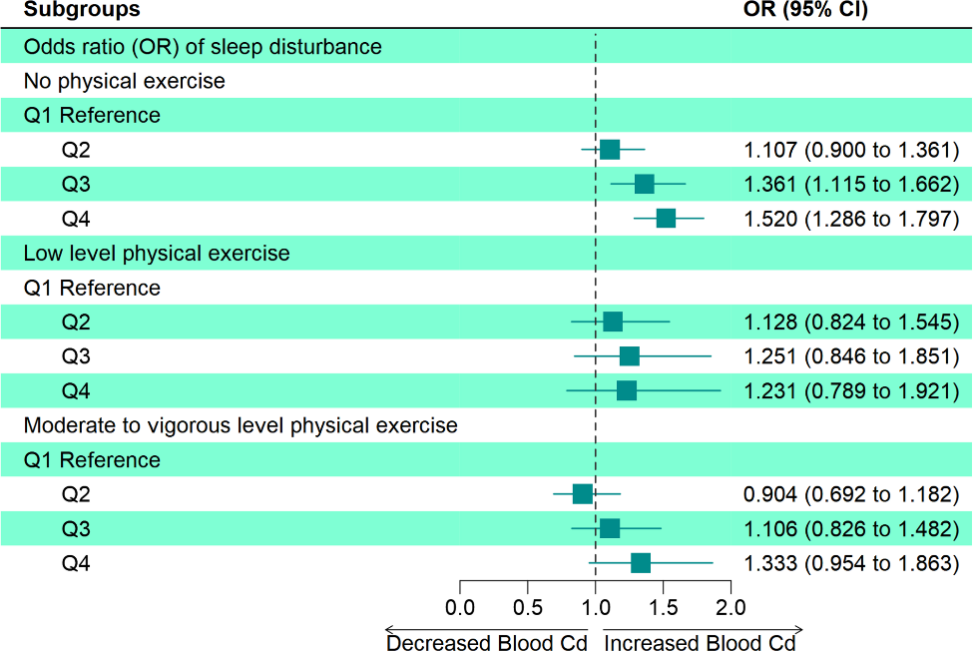
Association between blood Cd and sleep disturbance under different physical exercise levels

Moreover, subgroup analysis was conducted on the association between Cd exposure and sleep disturbance under different levels of PE (Fig. 3). In the association between Q4 of Cd exposure and sleep disturbance, the MVPE group (OR = 1.333, 95%CI: 0.954 ~ 1.863) had lower risks than the none exercise group (OR = 1.520, 95%CI: 1.286 ~ 1.797). Additionally, this trend was also identified in the relationship between Q3 level of blood Cd and sleep disturbance, that MVPE group (OR = 1.106) was associated with lower OR than the none exercise (OR = 1.361) and low exercise group (OR = 1.251). This indicated that in higher Cd exposure, maintaining moderate-to-vigorous exercise was associated with lower sleep disturbance rate. We also conducted analysis between PE volume and sleep disturbance under different levels of blood cadmium (Supplementary Fig. 2). In Cd = Q3, a significant association was observed between MVPE and sleep disturbance [OR (95% CI): 0.699 (0.533,0.916), p = 0.014].

**Fig. 4:**
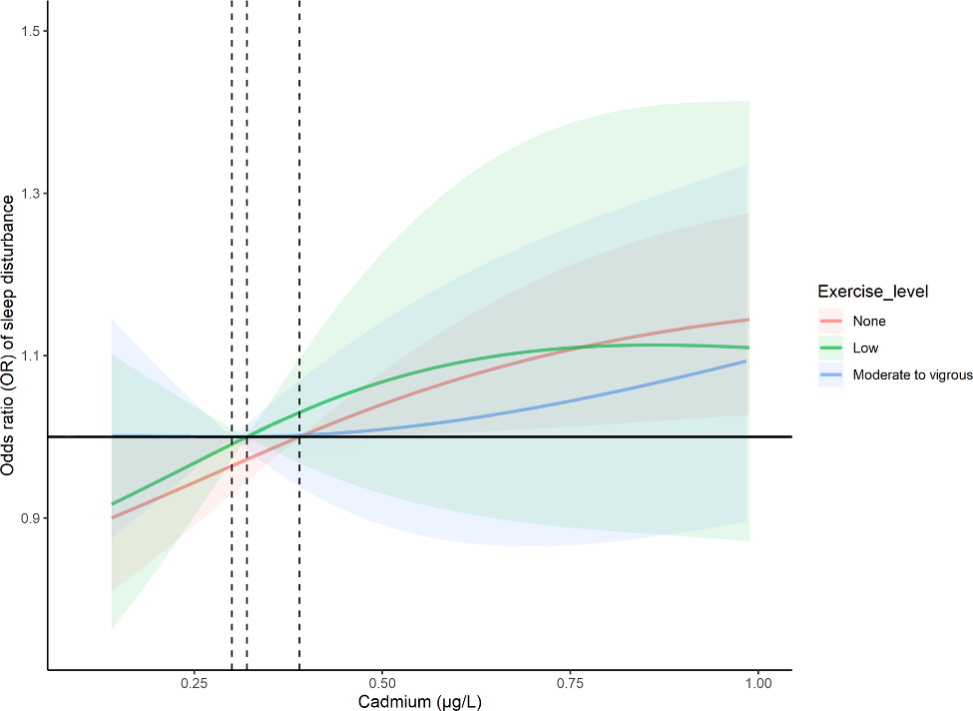
Dose-response relationship between blood Cd and sleep disturbance under different physical exercise levels

Based on the fully adjusted model, weighted restricted cubic spline was used to examine the dose-response relations between Cd exposure and sleep disturbance under different levels of PE (Fig. 4). It was found that blood Cd was positively associated with the incidence of sleep disturbance. In lower Cd exposure, there was not a significant trend between Cd level and sleep disturbance with different PE volume. However, with the increment in Cd concentration before it reached 1 µg/l, this relationship weakened with the increase in exercise volume, with the highest risk in the no exercise activity group, but the weakest risk in the MVPE group.

## Discussions

Using a representative cross-sectional survey, this study first identified that higher blood Cd exposure was significantly associated with sleep disturbance after adjustment for confounding factors. WQS model detected that the mixture effect of exposure to heavy metals was mainly attributable to Cd. Moreover, two-metal and multi-metal verified this association. However, no significant associations were found between Pb, Hg and risk of sleep disturbance. In addition, we not only elucidated negative effects of Cd exposure on sleep health, but also found countermeasures by doing more exercise to mitigate this association. Despite the fact that low level PE could not produce significant mitigation effects, MVPE could alleviate such associations in higher levels of Cd exposure. Further RCS model analysis verified these findings in a dose-response manager.

To date, our findings offered insights for the relationship between Cd exposure and the risk of sleep disturbance in the largest nationwide population. The impacts of Cd exposure on health outcomes such as cognition and mortality have been widely reported in previous studies [29–31], however the influence of Cd exposure on sleep health remained unknown. One animal study explored the effect of Cd exposure on brain structure and function in rats, results showed that one single injections of cadmium chloride lead to significant changes of wakefulness-sleep cycle [32]. Neurodegeneration due to the generation of reactive oxygen species (ROS) by other heavy metals, whether directly or not has been previously reported [33, 34]. From the mechanism, an increase and accumulation of ROS after Cd exposure might help to explain this phenomenon. Another rats study verified this hypothesis that Cd-induced sleep disturbance as a consequence of oxidative stress [35]. Considering that oxidized glutathione is one of endogenous sleep substances, Cd could induce abnormal sleep wake cycle by occupying intrinsic sleep-inducing neurotransmitters. In detail, the dysregulated activity of neurotransmitters involved in sleep regulation such as dopamine and serotonin [36] could be affected by high doses of metal exposure in the brain. Accounting for the facts that neurotransmitters regulating circadian rhythms also played important roles in neurodegenerative disorders [37], knowing the mechanism of Cd exposure on sleep might also help to prevent metal induced sleep problems and its related diseases.

Benefits of physical exercise on sleep status has received increasingly attention in recent years. Several epidemiological research reported the positive effects of exercise on sleep efficiency and quality [38–40], while the evidence on its rescue effects on heavy metal exposure was limited. According to our results, with the increment in Cd level, people performing regular MVPE were less likely to suffer from sleep disturbance. When it comes to the mechanism, inflammation and ROS and its related neurotransmitters may produce a marked effect on this process. Exercise-induced cytokine response, including predominant anti-inflammatory effect, may explain part of the improvement of sleep condition [41]. Early literature reported that long term regular PE can have an anti-inflammatory effect and reduce C- reaction protein levels [42]. Notably, the brain-derived neurotrophic factor (BDNF) as well as the glial cell-derived neurotrophic factor (GDNF) were other key factors induced by exercise. Studies have shown that exercise can strikingly upregulate the BDNF’s expression [43, 44]. As one of the most versatile neurotrophic factors in the brain, BDNF and GDNF played critical roles in the brain function, which mediated the protection of neurons, which in turn, could protect oxidative stress caused by ROS. Additionally, intensity mattered in the mitigation effects of metal exposure. In consideration of the cumulative effect of PE, previous publications suggested that chronic vigorous exercise was positively related to sleep quality enhancement. This was consistent with our findings that none or low level exercise was not enough to induce the rescue influence. Performing moderate to high level exercise could trigger secretions of multiple hormones in the neuro system, such as dopamine and serotonin [45], which were closely related to the ROS elimination.

This study reinforced the findings of adverse effects of heavy metals, especially Cd exposure on the sleep health. The strengths included the general-population study design, appreciable sample size, survey-weighted based analytical methods based on the complex multistage sampling design. Comprehensive baseline characteristics from the representative US adults’ sample enabled us to use multiple adjusted model for potential confounding factors. Moreover, compared with previous studies that only focused on the hazard effects of metals on human health, we not only verified the negative effects of Cd exposure, but also put forward a valid strategy, performing moderate to vigorous exercise, to mitigate sleep disturbances associated with Cd exposure.

Potential limitations should be acknowledged. First, although NHANES database only included three blood heavy metals in the 2007–2010 cycle, it was possible that there were still other metals with not available data may influence the results. Thus the findings of this study should be interpreted with caution due to the mixed-metal model only included three metals for analysis. Secondly, given the cross-sectional study design, a causal relationship between metal exposure and sleep disturbance cannot be confirmed. The assessment of sleep disturbance and physical exercise was based on the self-reported interview. This might cause report bias, although most previous studies tended to use such questionnaires rather than wearable fitness tracker devices [46]. Third, special groups such as industrial workers who were routinely at risk of heavy metal exposure, including those working in chemical plants, were worthy of further attention. Additionally, although several potential covariates including demographic and lifestyle factors in the analyses were adjusted, future studies should consider incorporating a wider array of chronic diseases and relevant comorbidity measures to capture a more comprehensive picture of sleep disturbance and its associations with metal exposures. Last but not least, other specific target population, mostly the child, teenager and pregnant woman did not receive strong evidence for reference. Intergenerational effect (e.g., influence of maternal exposure on offspring) of metal exposure and other rescue methods requested further validation.

## Conclusion

In summary, this study was the first to examine the relationship between blood Cd exposure and sleep disturbance in a nationwide population. Our results revealed the hazard impact of Cd exposure, highlighted the positive effects of exercise in mitigating such influence and expressed the underlying biology of pathways among these associations. This study indicated that lower Cd exposure and more PE were associated with a lower prevalence of sleep disturbance. Although our preliminary analysis found the association between blood Cd and sleep related disorder, such findings warranted biological investigations. Moreover, further studies employing longitudinal designs and objective measurements of sleep disturbance and physical exercise would provide more robust and specific insights into these relationships.

### Electronic supplementary material

Below is the link to the electronic supplementary material.

Supplementary Material 1

## Data Availability

(ADM) The datasets generated and analyzed for the current study are available in the NHANES repository. These data can be accessed using the following link: https://wwwn.cdc.gov/nchs/nhanes/Default.aspx.
