# Supplementary material for: Mitigation role of physical exercise participation in the relationship between blood cadmium and sleep disturbance: a cross-sectional study
Source: BMC Public Health. 2023 Jul 31;23:1465. doi: 10.1186/s12889-023-16358-4 (PMC10391747; doi:10.1186/s12889-023-16358-4)
Supplement: Supplementary file 1 — Supplementary Material 1 [file 12889_2023_16358_MOESM1_ESM.docx]

Supplementary file.


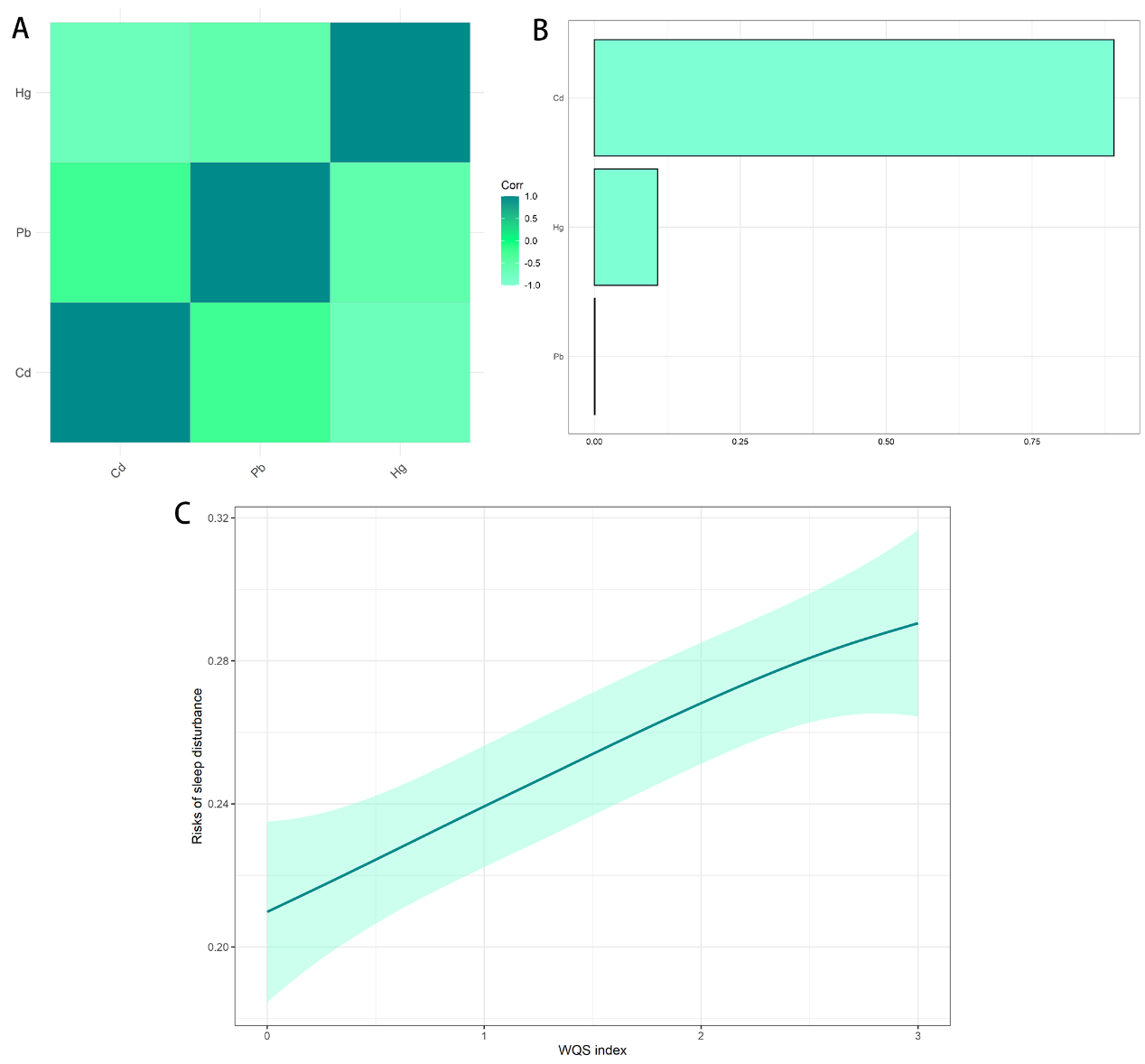


Figure S1. A) Correlations of all blood metals in NHANES, 2007–2010; B) Weights for weighted quantile sum regression index for mixed metal exposure; C) Smooth curve fitting for association between WQS index and risks of sleep disturbance. Fully adjusted model (Model 2) was used for analysis.


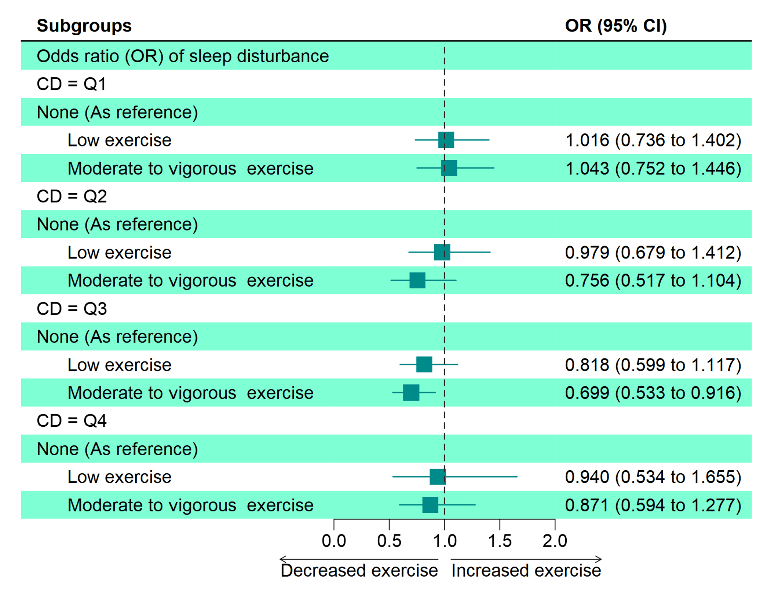


Figure S2. Association between PE level and sleep disturbance under different levels of blood Cd.

Supplementary Table 1. Weighted logistic regression results for blood Pb and Hg with sleep disturbance.

|  | Pb (µg/dl) | | | |
| --- | --- | --- | --- | --- |
|  | Q1 (< 0.89) | Q2 (0.89-1.37) | Q3 (1.37-2.13) | Q4 (> 2.13) |
| OR (95% CI) | Ref. | 0.996(0.842,1.177) | 0.972(0.767,1.231) | 0.929(0.715,1.205) |
| *P-value* |  | 0.219 | 0.423 | 0.071 |
|  | Hg (µg/l) | | | |
|  | Q1 (<0.49) | Q2 (0.49-0.88) | Q3 (0.88-1.68) | Q4 (>1.68) |
| OR (95% CI) | Ref. | 0.884(0.718,1.089) | 0.917(0.728,1.154) | 0.774(0.583,1.027) |
| *P-value* |  | 0.956 | 0.795 | 0.545 |

Notes: Fully adjusted model were used. Age, gender, race/ethnicity, body mass index, marital status, education, poverty income ratio, alcohol use, and chronic diseases were adjusted.

Supplementary Table 2. Weighted logistic regression of stratified results for blood Cd with sleep disturbance.

|  | Q1 | Q2 | *p* | Q3 | *p* | Q4 | *p* | p4trend | p4interaction |
| --- | --- | --- | --- | --- | --- | --- | --- | --- | --- |
| Gender |  |  |  |  |  |  |  |  | 0.544 |
| Male | Ref. | 0.998(0.781,1.275) | 0.984 | 1.192(0.915,1.552) | 0.185 | 1.240(0.936,1.643) | 0.128 | 0.09 |  |
| Female | Ref. | 0.992(0.814,1.207) | 0.930 | 1.186(0.966,1.456) | 0.101 | 1.509(1.185,1.923) | 0.002 | <0.001 |  |
| Age |  |  |  |  |  |  |  |  | 0.022 |
| < 40 | Ref. | 0.871(0.651,1.166) | 0.341 | 1.067(0.791,1.440) | 0.659 | 1.850(1.401,2.442) | <0.001 | <0.001 |  |
| [40, 60) | Ref. | 0.998(0.762,1.307) | 0.986 | 1.097(0.851,1.415) | 0.461 | 1.318(1.059,1.639) | 0.015 | 0.025 |  |
| ≥ 60 | Ref. | 0.875(0.623,1.228) | 0.426 | 1.039(0.737,1.464) | 0.824 | 0.858(0.593,1.241) | 0.403 | 0.689 |  |
| Race/ethnicity |  |  |  |  |  |  |  |  | 0.362 |
| non-Hispanic White | Ref. | 1.025(0.863,1.218) | 0.768 | 1.279(1.010,1.619) | 0.042 | 1.419(1.189,1.694) | <0.001 | <0.001 |  |
| non-Hispanic Black | Ref. | 1.297(0.804,2.092) | 0.274 | 1.388(0.920,2.093) | 0.113 | 1.501(1.001,2.251) | 0.049 | 0.032 |  |
| Mexican American | Ref. | 1.688(1.012,2.815) | 0.045 | 1.727(1.159,2.573) | 0.009 | 2.383(1.490,3.810) | <0.001 | <0.001 |  |
| Other Race/ethnicity | Ref. | 0.709(0.479,1.049) | 0.083 | 1.123(0.676,1.866) | 0.643 | 1.292(0.811,2.057) | 0.269 | 0.136 |  |
| Marital status |  |  |  |  |  |  |  |  | 0.035 |
| Never married | Ref. | 1.264(0.888,1.799) | 0.186 | 1.425(0.995,2.042) | 0.053 | 1.651(1.118,2.439) | 0.014 | 0.011 |  |
| Married/living with partner | Ref. | 0.968(0.797,1.177) | 0.738 | 1.352(1.059,1.726) | 0.017 | 1.454(1.162,1.819) | 0.002 | <0.001 |  |
| Widowed/ divorced | Ref. | 0.899(0.628,1.287) | 0.549 | 0.721(0.490,1.062) | 0.095 | 0.907(0.607,1.356) | 0.625 | 0.597 |  |
| Poverty income ratio. |  |  |  |  |  |  |  |  | 0.008 |
| < 1 | Ref. | 1.198(0.736,1.948) | 0.454 | 1.620(1.209,2.171) | 0.002 | 2.370(1.585,3.544) | <0.001 | <0.001 |  |
| [1,3) | Ref. | 1.068(0.800,1.426) | 0.645 | 1.534(1.087,2.165) | 0.017 | 1.761(1.365,2.273) | <0.001 | <0.001 |  |
| ≥ 3 | Ref. | 1.010(0.842,1.211) | 0.912 | 1.106(0.810,1.509) | 0.513 | 1.061(0.823,1.367) | 0.638 | 0.546 |  |
| Education |  |  |  |  |  |  |  |  | 0.134 |
| Below high school | Ref. | 1.777(1.116,2.828) | 0.017 | 2.016(1.303,3.121) | 0.003 | 3.513(2.059,5.996) | <0.001 | <0.001 |  |
| High school | Ref. | 0.965(0.741,1.257) | 0.784 | 1.116(0.810,1.540) | 0.489 | 1.204(0.881,1.647) | 0.234 | 0.172 |  |
| College or above | Ref. | 1.048(0.842,1.303) | 0.667 | 1.341(1.026,1.753) | 0.033 | 1.551(1.194,2.014) | 0.002 | 0.002 |  |
| BMI(kg/m^2^) |  |  |  |  |  |  |  |  | 0.096 |
| < 25 | Ref. | 0.996(0.661,1.501) | 0.985 | 1.332(0.912,1.947) | 0.133 | 1.821(1.320,2.513) | <0.001 | <0.001 |  |
| [25, 30) | Ref. | 0.983(0.748,1.292) | 0.898 | 1.504(1.147,1.973) | 0.005 | 1.652(1.258,2.170) | <0.001 | <0.001 |  |
| ≥ 30 | Ref. | 1.189(0.918,1.540) | 0.181 | 1.173(0.909,1.515) | 0.211 | 1.263(0.946,1.687) | 0.109 | 0.121 |  |
| Smoking status |  |  |  |  |  |  |  |  | 0.072 |
| None | Ref. | 0.934(0.814,1.072) | 0.321 | 1.198(0.952,1.508) | 0.119 | 1.050(0.777,1.418) | 0.744 | 0.238 |  |
| Former smoking | Ref. | 1.168(0.829,1.647) | 0.361 | 1.226(0.854,1.761) | 0.259 | 1.050(0.738,1.492) | 0.780 | 0.587 |  |
| Current smoking | Ref. | 1.822(0.575,5.769) | 0.296 | 2.061(0.762,5.568) | 0.148 | 2.854(1.084,7.510) | 0.035 | 0.002 |  |
| Alcohol use |  |  |  |  |  |  |  |  | 0.012 |
| None | Ref. | 0.958(0.694,1.321) | 0.785 | 1.409(1.008,1.968) | 0.045 | 1.713(1.237,2.372) | 0.002 | <0.001 |  |
| Moderate alcohol use | Ref. | 0.949(0.762,1.182) | 0.630 | 1.014(0.782,1.316) | 0.912 | 1.136(0.898,1.437) | 0.276 | 0.350 |  |
| High alcohol use | Ref. | 1.569(0.957,2.572) | 0.072 | 2.173(1.498,3.152) | <0.001 | 2.433(1.697,3.486) | <0.001 | <0.001 |  |
| CVD |  |  |  |  |  |  |  |  | 0.693 |
| No | Ref. | 0.921(0.465,1.825) | 0.808 | 0.945(0.584,1.529) | 0.812 | 1.319(0.725,2.401) | 0.352 | 0.238 |  |
| Yes | Ref. | 1.045(0.922,1.185) | 0.474 | 1.258(1.022,1.548) | 0.031 | 1.394(1.176,1.652) | <0.001 | <0.001 |  |
| DM |  |  |  |  |  |  |  |  | 0.473 |
| No | Ref. | 1.097(0.961,1.253) | 0.163 | 1.326(1.090,1.614) | 0.006 | 1.500(1.249,1.803) | <0.001 | <0.001 |  |
| Yes | Ref. | 0.828(0.511,1.340) | 0.429 | 0.989(0.647,1.511) | 0.956 | 1.130(0.763,1.673) | 0.529 | 0.338 |  |
| Physical exercise |  |  |  |  |  |  |  |  | 0.740 |
| None | Ref. | 1.107(0.900,1.361) | 0.324 | 1.361(1.115,1.662) | 0.004 | 1.520(1.286,1.797) | <0.001 | <0.001 |  |
| Low | Ref. | 1.128(0.824,1.545) | 0.440 | 1.251(0.846,1.851) | 0.251 | 1.231(0.789,1.921) | 0.346 | 0.279 |  |
| Moderate to vigorous | Ref. | 0.904(0.692,1.182) | 0.449 | 1.106(0.826,1.482) | 0.484 | 1.333(0.954,1.863) | 0.090 | 0.095 |  |

Notes: Q1 of blood Cd level was used as reference. Abbreviations: BMI, body mass index; PIR, poverty income ratio; Cd, cadmium; DM, diabetes mellitus; CVD, cardiovascular diseases; p4trend, p for trend; p4interaction, p for interaction.
